# Approach Coping Mitigates Distress of COVID-19 Isolation for Young Men With Low Well-Being in a Sample of 1,749 Youth From Australia and the USA

**DOI:** 10.3389/fpsyt.2021.634925

**Published:** 2021-04-28

**Authors:** Phillip (Xin) Cheng, Haeme R. P. Park, Justine M. Gatt

**Affiliations:** ^1^Neuroscience Research Australia (NeuRA), Sydney, NSW, Australia; ^2^School of Psychology, University of New South Wales (UNSW), Sydney, NSW, Australia

**Keywords:** COVID-19, isolation, wellbeing, coping, psychological impact & pandemic, adolescents, youth-young adults, COMPAS-W

## Abstract

The unprecedented COVID-19 pandemic has led to lockdowns across the world with people being separated from their loved ones including partners, family, and friends. Here, using a large sample of 1,749 Australians and Americans, we investigated the impact of COVID-19 isolation on younger populations (13–25 years), and the influence of coping strategies and mental well-being on this impact. Overall, COVID-19 isolation had a more negative impact on adolescence (13–17 years) than young adulthood (18–25 years), but with no difference apparent between men and women, or between Australian and American residents. However, a deeper analysis revealed a gender-specific effect: the type of coping strategies differentially influenced the negative impact of COVID-19 isolation on men with various levels of well-being, an interaction effect not apparent in women. For men with lower levels of mental well-being, COVID-19 isolation appeared to have a less negative impact on them if they used more approach-oriented coping strategies (e.g., actively focusing on the problem). Our results provide cross-sectional evidence for a differential impact on young men at low levels of wellbeing by pandemic isolation. In sum, young men and adolescent boys with lower well-being coped better with COVID-19 isolation when they used more approach coping strategies.

## Introduction

The COVID-19 pandemic has drastically changed our lives. People across the world have been isolated for months or more, and physical contact with individuals outside of their homes has typically been discouraged or prohibited. Isolation often means separation from loved ones including family, friends, or partners, which can result in an increase in distress in any population (1–5), and exacerbate distress particularly in younger populations [e.g., 16–25 years (6)]. Although numerous studies have examined the psychological impact of COVID-19 and preventive measures on general populations [e.g., (7–11)], few have done so within younger cohorts. It is therefore crucial to identify the factors that might mitigate the distress of COVID-19 isolation on younger populations.

A major factor that may contribute to the extent to which one is negatively affected by isolation is the ability to maintain optimal well-being. Well-being can be defined as a combination of both subjective and psychological well-being; for instance, happiness, life satisfaction, positive relations with others, and goal-directed behavior (12). Research has reliably shown an increase in negative symptoms such as stress and anxiety for people with low well-being (13–17). Recent studies investigating the impact of the COVID-19 pandemic on mental well-being have corroborated that adults who reported higher well-being as the lockdown continued exhibited reduced psychiatric symptoms (18) and improved overall work performance (19). Accordingly, maintaining a healthy level of well-being is important as it may buffer against potential vulnerability to illness following adversity (20). Here, we examine the relationship between well-being and impacts of COVID-19 isolation in a large sample of 1,749 adolescents and young adults residing in Australia and the USA.

Another potential predictor for the level of distress during the pandemic is the type of coping strategies used under stressful situations. Previous studies have shown that people who rely on avoidance-oriented coping strategies (e.g., self-distraction) have a heightened probability of exhibiting psychiatric symptoms compared to people relying on active coping styles such as active problem solving (21, 22). Consistent are the findings that following 3 months of SARS quarantine, healthcare workers who reported higher levels of stress and anxiety also exhibited increased engagement in avoidance behavior [i.e., reduced contact with other people and avoiding crowded places (23)]. During the COVID-19 lockdown, individuals engaging in avoidance behavior (e.g., alcohol consumption and excessive eating) exhibit higher levels of anxiety (24) and distress (25) than those using active coping strategies (e.g., maintaining social communication). Other research has highlighted the importance of active coping in reducing panic and other negative feelings and improving well-being in times of stress (26–31). Together, these findings indicate that adopting an approach-oriented coping mechanism (i.e., actively focusing on challenges) could help people alleviate distress during a pandemic, as well as other potential stressors.

In the current study, we will explore how well-being and the type of coping strategies interact in influencing the impact of COVID-19 isolation on a sample of 1,749 community participants aged from 13 to 25 years across Australia and the USA. Here we focus on the avoidance-oriented and approach-oriented coping styles. We expect that individuals with higher well-being should be more resilient to the negative impact of isolation than people with lower well-being. In addition, because avoidance behavior has been linked with increased psychiatric symptoms after isolation, whereas maintaining social connectedness is associated with reduced negative feelings, we predict that adopting a more active coping style would help reduce the negative impact of isolation. We therefore predict that approach coping strategies would benefit individuals who demonstrate lower levels of well-being in particular, in terms of coping with the effect of separation. Finally, since previous findings are mixed regarding whether demographics is predictive of the psychological outcomes of isolation (6, 32), we will also consider the modulating influence of gender, age, and country of residence on these relationships.

## Methods

### Participants

Survey data was collected in a sample of 2,580 younger participants (13–25 years) *via* Qualtrics survey panels of community participants residing in Australia or the USA. The Qualtrics Team sent database members of online Qualtrics survey on our behalf to selected members. Participant recruitment focused on three defining criteria: age (13–17 years and 18–25 years, 50:50), gender (men and women, 50:50), and country of residence (Australia and USA, 50:50). Inclusion criteria included English as a primary language. Parental/guardian consent for 13–17 year old participants was first obtained using the opt-out approach, followed by implied consent of the participant when they completed the survey. Participants were asked to complete a 20-min survey on four occasions (every 6 weeks) over a 6-month period, although they were able to opt out of the study at any time. For Qualtrics panels, all of the participants are assured confidentiality, and they are anonymous to the researchers. To exclude duplication and ensure validity, Qualtrics checks every IP address and uses a sophisticated digital fingerprint technology. In addition, every strategic panel partner uses deduplication technology to provide the most reliable results and retain the integrity of the survey data. They adopt Relevant ID in addition to this which has the unique ability to identify multiple panel accounts from different research firms on the same computer. Suspect respondents are flagged in the system and are either allowed, redirected or completely filtered out of surveys in which they attempt to participate. The Qualtrics Team reimbursed participants for their participation by means of a small incentive (e.g., points or gift vouchers) per survey completion. The study was approved by the Human Research Ethics Committee of the University of New South Wales (UNSW), Sydney, Australia (HC200150).

For the current study, to ensure our analyses only included participants who accurately completed the surveys, we observed responses to key items and removed those participants (*n* = 831) who provided positive-impact ratings for events that should have been rated as negative (e.g., death/severe illness of the immediate family). The final sample included 1,749 participants ranging in age from 13 to 25 years (M = 17.76; SD = 3.72), 656 men/1,093 women, of which 944 participants resided in Australia and 805 in the US (Table 1).

**Table 1:** Number of participants by demographic variables.

**Demographic variables**	**Categories**	**Number of participants (%)**
Gender	Female	1,093 (62.49)
	Male	656 (37.51)
Age	Adolescents (13–17 years)	1,062 (60.72)
	Young adults (18–25 years)	687 (39.28)
Country of residence	Australia	944 (53.97)
	US	805 (46.03)
Marital status	Single	1,438 (82.22)
	In a relationship	225 (12.86)
	Married/de facto	86 (4.92)
Employment	Employed	508 (29.05)
	Unemployed	1,175 (67.18)
	Unanswered	66 (3.77)
History of psychological disorders	No	1,501 (85.82)
	Yes	248 (14.18)
Family income	None	38 (2.17)
	$1–$15,000	97 (5.55)
	$15,001–$30,000	101 (5.77)
	$30,001–$50,000	70 (4.00)
	$50,001–$75,000	94 (5.37)
	$75,001–$100,000	65 (3.72)
	$100,001–$125,000	31 (1.77)
	$125,001–$150,000	19 (1.09)
	$150,001–$200,000	30 (1.72)
	$200,001–$250,000	9 (0.51)
	$250,001–$300,000	6 (0.34)
	$300,001 and over	21 (1.20)
	Prefer not to answer	60 (3.43)
	Unanswered	1,108 (63.35)

### Measures and Procedures

The data for the current study was based on the baseline data collected *via* the Qualtrics Survey Panels. Baseline data was collected from 1st May to 10th June 2020. Participants used their own digital devices to complete the surveys. We drew on survey data that measured mental well-being, coping strategies under stressful situations, and the impact of COVID-related isolation from partners, friends and family.

The 26-item COMPAS-W scale was used to measure well-being (12). The COMPAS-W has been validated in adults (12) and adolescents aged 12 years and above (33), and has been employed in several previous psychometric, neuropsychological and RCT studies with well-being as the outcome [e.g., (16, 34–40)]. It provides a composite measurement of both subjective and psychological well-being, as well as six well-being sub-dimensions: Composure (e.g., “When I'm faced with a stressful situation, I usually make myself think about it in a way that helps me stay calm.”), Own worth (e.g., “I often get upset at the way people treat me.”), Mastery (e.g., “When something is going to affect me, I usually learn as much about it as I can”), Positivity (e.g., “I am usually quite a happy and positive person.”), Achievement (e.g., “I have a clear set of goals and work toward them in an orderly fashion.”), and Satisfaction (e.g., “I would rate my quality of life as very good.”). This scale has a high internal reliability (0.84) and high test-retest reliability [0.82 (12)]. Total scores were used to quantify well-being.

The 28-item Brief-COPE scale was used to measure ways of coping during stressful life events (41). The Brief-COPE broadly categorizes coping strategies into Avoidance coping and Approach coping. Avoidance coping contains six subscales: denial (e.g., “I've been saying to myself ‘this isn't real.”'), substance use (e.g., “I've been using alcohol or other drugs to make myself feel better.”), venting (e.g., “I've been saying things to let my unpleasant feelings escape.”), behavioral disengagement (e.g., “I've been giving up trying to deal with it.”), self-distraction (e.g., “I've been turning to work or other activities to take my mind off things.”) and self-blame (e.g., “I've been criticizing myself.”) [see (42) for a review]. Approach coping contains six subscales: active coping (e.g., “I've been concentrating my efforts on doing something about the situation I'm in.”), positive reframing (e.g., “I've been trying to see it in a different light, to make it seem more positive.”), planning (e.g., “I've been trying to come up with a strategy about what to do.”), acceptance (e.g., “I've been accepting the reality of the fact that it has happened.”), seeking emotional support (e.g., “I've been getting emotional support from others.”), and seeking informational support (e.g., “I've been getting help and advice from other people.”).^1^ The Brief-COPE subscales have an internal reliability ranging from 0.50 minimum to 0.90 and a test-retest reliability over 0.55 [e.g., (41, 43–47)]. Total scores were used to quantify the likelihood of adopting a particular coping strategy under stressful situations.

To study the impact of COVID-19 isolation, we asked participants whether they were separated from loved ones due to the pandemic using the following three questions: “Did you experience: (1) Temporary separation from girlfriend/boyfriend due to COVID-19?; (2) Temporary separation from your close friends due to COVID-19?; and (3) Temporary separation from your immediate family due to COVID-19?”. If the answer was yes to any of these questions, the participants then rated the positive vs. negative impact this experience had on their life using a 5-point scale (−2: extremely negative; −1: somewhat negative; 0: neutral; 1: somewhat positive; 2: extremely positive). Mean scores were used to quantify the impact of isolation.

### Analyses

To examine how younger people's well-being, coping strategies and demographics affect their experience during the COVID-19 isolation, we tested whether the variance explained by these variables were significantly greater than the unexplained variance using omnibus regression models (*Predictors*: COMPAS-W scores, Brief-COPE scores (Avoidance/Approach), age, gender, country, and the interactions of these factors; *Outcome*: COVID-19 isolation impact ratings). In the statistical analysis, age, gender, and country of residence were included as predictors, and we also controlled for the effect of marital status, employment, family income, and history of psychological disorders in all models. In our analyses, we treated age as a categorical variable with two levels based on developmental periods: adolescence (13–17 years) and young adulthood (18–25 years). Linear mixed-models were used to explore any significant effects found for the predictor variables. Multiple comparisons were Bonferroni-corrected for significant interaction effects (*p* < 0.008). All analyses were two-tailed. Statistics were performed using MATLAB R2020a.

## Results

We first examined the level of well-being during the COVID-19 pandemic for our sample as a function of age, gender, and country of residence. The mean COMPAS-W well-being scores were 96.18 (SD = 13.95) for adolescents (13–17 years) and 88.48 (SD = 14.28) for young adults (18–25 years); 91.85 (SD = 14.45) for women and 95.33 (SD = 14.51) for men; and 92.49 (SD = 13.92) for Australians and 93.93 (SD = 15.27) for Americans. Using a Bonferroni-corrected significance threshold of 0.017 (0.05/3), two-sample *t*-tests showed that (1) adolescents scored significantly higher on COMPAS-W than young adults [*t*_(1, 747)_ = 10.59, *p* < 0.001]; (2) male participants scored significantly higher on COMPAS-W than female participants [*t*_(1, 747)_ = 5.06, *p* < 0.001]; with (3) no significant difference between Australians and Americans [*t*_(1, 747)_ = 1.76, *p* = 0.078] for well-being.

### Associations Between Well-Being and Coping Strategies

Next we checked the relationship between well-being and coping strategies. According to the findings of previous studies, the tendency of using Avoidance coping is associated with negative psychological outcomes, whereas the tendency of using Approach coping is associated with positive psychological outcomes (42). Consistent with previous findings, we found a significant negative correlation between COMPAS-W well-being scores and Avoidance coping scores [*r*_(1, 747)_ = −0.35; *p* < 0.001], and a significant positive correlation between COMPAS-W well-being scores and Approach coping scores [*r*_(1, 747)_ = 0.10; *p* < 0.001]. This suggests individuals who use more approach coping strategies tend to have higher well-being, whereas those who use more avoidance coping strategies tend to have lower well-being.

### Coping Strategies by Age, Gender, and Country of Residence

Whether the coping strategies individuals used differ by their age, gender, and country of residence was then examined. Using a Bonferroni-corrected significance level of 0.017 (0.05/3), adolescents (M = 19.16, SD = 6.70) used significantly less Avoidance coping than young adults [M = 24.11, SD = 7.57; *t*_(1, 747)_ = −14.24, *p* < 0.001]. Adolescents also used significantly less Approach coping than young adults [adolescents: M = 26.78, SD = 9.00; young adults: M = 29.30, SD = 8.22; *t*_(1, 749)_ = −5.89, *p* < 0.001]. Women (M = 21.44, SD = 7.16) used significantly more Avoidance coping than men [M = 20.55, SD = 7.89; *t*_(1, 747)_ = 2.42, *p* = 0.015], and their use of Approach coping did not differ significantly [women: M = 28.10, SD = 8.49; men: M = 27.23, SD = 9.24; *t*_(1, 747)_ = 1.82, *p* = 0.069]. The use of Avoidance coping did not differ significantly between Australians (M = 20.80, SD = 7.60) and Americans (M = 21.47, SD = 7.27; *t*_(1, 747)_ = −1.81, *p* = 0.070). Americans (M = 28.45, SD = 8.80) used significantly more Approach coping than Australians [M = 27.19, SD = 8.74; *t*_(1, 747)_ = 3.12, *p* = 0.002].

### The Negative Impact of COVID-19 Isolation

We next examined the general impact of COVID-19 isolation (using an average of responses to the three COVID-19 separation questions) relative to the baseline of zero (indicating “no impact”) on our sample. A one-sample *t*-test showed that the sample mean rating of −0.63 (SD = 0.76) was significantly lower than zero [*t*_(1, 555)_ = −58.62, *p* < 0.001], suggesting that COVID-19 isolation indeed had a negative impact on younger people's lives overall.

### The Effect of COVID-19 Isolation by Age, Gender, and Country of Residence

To check whether COVID-19 isolation had a differential impact on different demographic groups, we separately analyzed age, gender, and country of residence. Using a Bonferroni-corrected significance threshold of 0.017 (0.05/3), both age groups rated COVID-19 isolation as being a negative experience (adolescents: M = −0.68, SD = 0.76; young adults: M = −0.54, SD = 0.75), but the impact was significantly more negative for adolescents [*t*_(1, 554)_ = −3.54, *p* < 0.001]. Furthermore, although men (M = −0.60, SD = 0.77) and women (M = −0.65, SD = 0.75) rated COVID-19 isolation as a negative experience, the ratings did not differ significantly between them [*t*_(1, 554)_ = 1.30, *p* = 0.193]. Finally, both Australians (M = −0.64, SD = 0.73) and Americans (M = −0.62, SD = 0.79) rated COVID-19 isolation as having a negative impact, but again the impact did not differ significantly between the two countries [*t*_(1, 554)_ = −0.53, *p* = 0.594]. These results suggest that COVID-19 isolation has a negative impact on individuals regardless of their demographics, and that it appears to more negatively affect adolescents than young adults.

### The Effect of Coping Strategies on the Impact of COVID-19 Isolation at Various Levels of Well-Being

We next examined how the impact of isolation varies as a function of people's well-being, coping strategies, and demographics. An omnibus regression model (Table 2) showed that the interaction of COMPAS-W well-being scores, Avoidance coping, age, gender, and country of residence was not significant (β < −0.001, *p* = 0.622). Similarly, there was no significant interaction of COMPAS-W well-being scores, Approach coping, age, gender, and country of residence (β = 0.002, *p* = 0.086; Table 3). Therefore, we decided to examine age, gender and country of residence separately. At this level, the interaction of COMPAS-W well-being scores, coping, and age was not significant (Avoidance: β < 0.001, *p* = 0.385; Approach: β < −0.001, *p* = 0.809). The interaction of COMPAS-W well-being scores, coping and country of residence was also not significant (Avoidance: β < 0.001, *p* = 0.597; Approach: β < 0.001, *p* = 0.703). The interaction of COMPAS-W well-being scores, Avoidance coping and gender was again not significant (β < −0.001, *p* = 0.606), but that of COMPAS-W well-being scores, Approach coping and gender was marginally significant (β = −0.001, *p* = 0.055; Table 3).

**Table 2:** Omnibus regression analysis with avoidance coping.

**Effects**	**β**	***SE***	**95% CI**		***p***
			**LL**	**UL**	
(Intercept)	−0.94	1.03	−2.97	1.09	0.365
Age	1.38	1.51	−1.57	4.34	0.359
Gender	−1.78	1.37	−4.47	0.91	0.194
Country of residence	0.52	1.56	−2.54	3.57	0.741
Avoidance	0.01	0.05	−0.08	0.10	0.826
COMPAS	0.00	0.01	−0.02	0.02	0.884
Age:Gender	0.30	2.46	−4.53	5.13	0.903
Age:Country of residence	−3.70	2.21	−8.03	0.63	0.094
Gender:Country of residence	2.17	2.28	−2.31	6.64	0.342
Age:Avoidance	−0.06	0.06	−0.18	0.07	0.363
Gender:Avoidance	0.05	0.06	−0.07	0.17	0.452
Country of residence:Avoidance	−0.04	0.07	−0.18	0.10	0.557
Age:COMPAS	−0.01	0.02	−0.04	0.02	0.422
Gender:COMPAS	0.02	0.01	−0.01	0.05	0.229
Country of residence:COMPAS	0.00	0.02	−0.04	0.03	0.777
Avoidance:COMPAS	0.00	0.00	0.00	0.00	0.873
Age:Gender:Country of residence	−0.23	3.93	−7.95	7.48	0.953
Age:Gender:Avoidance	0.00	0.10	−0.20	0.21	0.975
Age:Country of residence:Avoidance	0.17	0.09	−0.01	0.36	0.067
Gender:Country of residence:Avoidance	−0.06	0.10	−0.26	0.15	0.570
Age:Gender:COMPAS	−0.01	0.03	−0.06	0.04	0.710
Age:Country of residence:COMPAS	0.04	0.02	−0.01	0.08	0.112
Gender:Country of residence:COMPAS	−0.02	0.02	−0.07	0.03	0.379
Age:Avoidance:COMPAS	0.00	0.00	0.00	0.00	0.385
Gender:Avoidance:COMPAS	0.00	0.00	0.00	0.00	0.606
Country of residence:Avoidance:COMPAS	0.00	0.00	0.00	0.00	0.597
Age:Gender:Country of residence:Avoidance	0.04	0.17	−0.28	0.37	0.771
Age:Gender:Country of residence:COMPAS	0.02	0.04	−0.07	0.10	0.697
Age:Gender:Avoidance:COMPAS	0.00	0.00	0.00	0.00	0.884
Age:Country of residence:Avoidance:COMPAS	0.00	0.00	0.00	0.00	0.094
Gender:Country of residence:Avoidance:COMPAS	0.00	0.00	0.00	0.00	0.671
Age:Gender:Country of residence:Avoidance:COMPAS	0.00	0.00	0.00	0.00	0.622

*CI, confidence interval; LL, lower limit; UL, upper limit*.

**Table 3:** Omnibus regression analysis with approach coping.

**Effects**	**β**	***SE***	**95% CI**		***p***
			**LL**	**UL**	
(Intercept)	−0.29	1.09	−2.43	1.84	0.770
Age	−0.66	1.59	−3.79	2.46	0.695
Gender	−3.69	1.47	−6.57	−0.81	0.014^*^
Country of residence	−0.08	1.61	−3.23	3.07	0.949
Approach	−0.02	0.04	−0.09	0.06	0.664
COMPAS	−0.00	0.01	−0.02	0.02	0.951
Age:Gender	2.22	2.59	−2.86	7.31	0.391
Age:Country of residence	−2.66	2.27	−7.11	1.79	0.242
Gender:Country of residence	1.79	2.29	−2.69	6.28	0.432
Age:Approach	0.02	0.06	−0.09	0.13	0.660
Gender:Approach	0.12	0.05	0.02	0.22	0.023^*^
Country of residence:Approach	−0.01	0.06	−0.12	0.10	0.889
Age:COMPAS	0.01	0.02	−0.03	0.04	0.714
Gender:COMPAS	0.03	0.01	0.00	0.06	0.022^*^
Country of residence:COMPAS	−0.00	0.02	−0.03	0.03	0.861
Approach:COMPAS	0.00	0.00	−0.00	0.00	0.947
Age:Gender:Country of residence	5.83	3.93	−1.88	13.53	0.138
Age:Gender:Approach	−0.04	0.09	−0.22	0.14	0.647
Age:Country of residence:Approach	0.11	0.08	−0.05	0.26	0.179
Gender:Country of residence:Approach	−0.05	0.08	−0.20	0.11	0.548
Age:Gender:COMPAS	−0.02	0.03	−0.08	0.03	0.402
Age:Country of residence:COMPAS	0.03	0.02	−0.01	0.08	0.166
Gender:Country of residence:COMPAS	−0.02	0.02	−0.06	0.03	0.496
Age:Approach:COMPAS	−0.00	0.00	−0.00	0.00	0.809
Gender:Approach:COMPAS	−0.00	0.00	−0.00	0.00	0.055
Country of residence:Approach:COMPAS	0.00	0.00	−0.00	0.00	0.703
Age:Gender:Country of residence:Approach	−0.17	0.13	−0.44	0.09	0.192
Age:Gender:Country of residence:COMPAS	−0.08	0.04	−0.16	0.01	0.065
Age:Gender:Approach:COMPAS	0.00	0.00	−0.00	0.00	0.711
Age:Country of residence:Approach:COMPAS	−0.00	0.00	−0.00	0.00	0.118
Gender:Country of residence:Approach:COMPAS	0.00	0.00	−0.00	0.00	0.658
Age:Gender:Country of residence:Approach:COMPAS	0.00	0.00	−0.00	0.01	0.086

*CI, confidence interval; LL, lower limit; UL, upper limit*;

* *Statistically significant p-value*.

**Figure 1:**
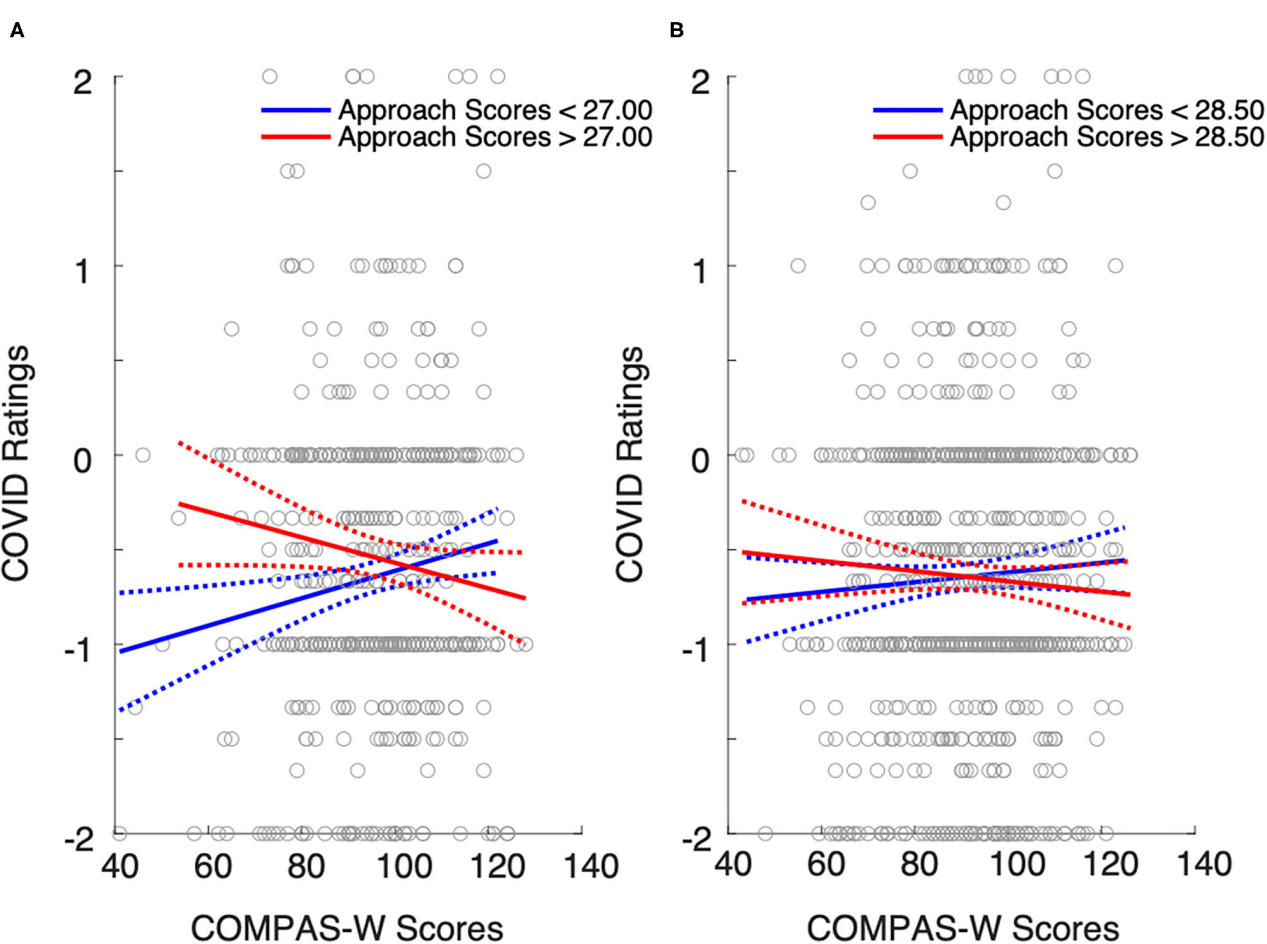
The effect of well-being and Approach coping on the impact of COVID isolation on **(A)** men and **(B)** women aged 13–25 years. The blue lines are the least-square regression fits for Approach coping scores below the sample median. The red lines are the least-square regression fits for Approach coping scores above the median. For male participants whose well-being level is low, the employment of Approach coping seems to reduce the negative impact of isolation. This effect does not occur for male participants with high well-being. For female participants, the use of Approach coping does not seem to affect the impact of isolation regardless of their level of well-being. Gray dots represent individual scores for COMPAS-W and COVID ratings. Dotted lines represent 95% confidence intervals.

We followed up the marginal interaction effect by separating the gender groups to examine whether the male or female sample (or both) contributed to the influence of well-being and approach coping strategies on the impact of COVID-19 isolation (Figure 1). The Bonferroni-corrected significance threshold was 0.025 (0.05/2). The results of one-way ANOVAs showed significant interaction effects between COMPAS-W well-being and Approach coping for men (β < −0.001, *p* = 0.002; Figure 1A) but not for women (β < −0.001, *p* = 0.317; Figure 1B). This suggests that COVID-19 isolation seems to have a differential impact on younger men depending on their use of Approach coping and levels of well-being. By contrast, the impact of COVID-19 isolation on younger women did not significantly vary with their well-being levels and coping styles.

To dissect the interaction of well-being and Approach coping, we used the medians of COMPAS-W and Brief-COPE (Approach) scores to classify the male sample into four groups: low-coping-low-well-being (LCLW), high-coping-low-well-being (HCLW), low-coping-high-well-being (LCHW), and high-coping-high-well-being (HCHW) groups. Figure 1A shows that when well-being is low, the impact of isolation appears to differ between high and low users of Approach coping, but this effect does not seem to occur when well-being scores are high. *Post-hoc* comparisons revealed that COVID-19 isolation impact ratings were significantly lower in the LCLW group compared to HCLW groups (β = 0.30, *p* < 0.001). By contrast, there was no significant difference in isolation ratings for LCHW and HCHW groups (β = −0.10, *p* = 0.245). These results suggest that the pandemic seems to have a more negative impact on younger men who do not seem to have an effective coping strategy, but only for those with a lower level of well-being.

### The Effect of Specific Approach Coping Strategies on the Impact of COVID-19 Isolation at Various Levels of Well-Being

**Figure 2:**
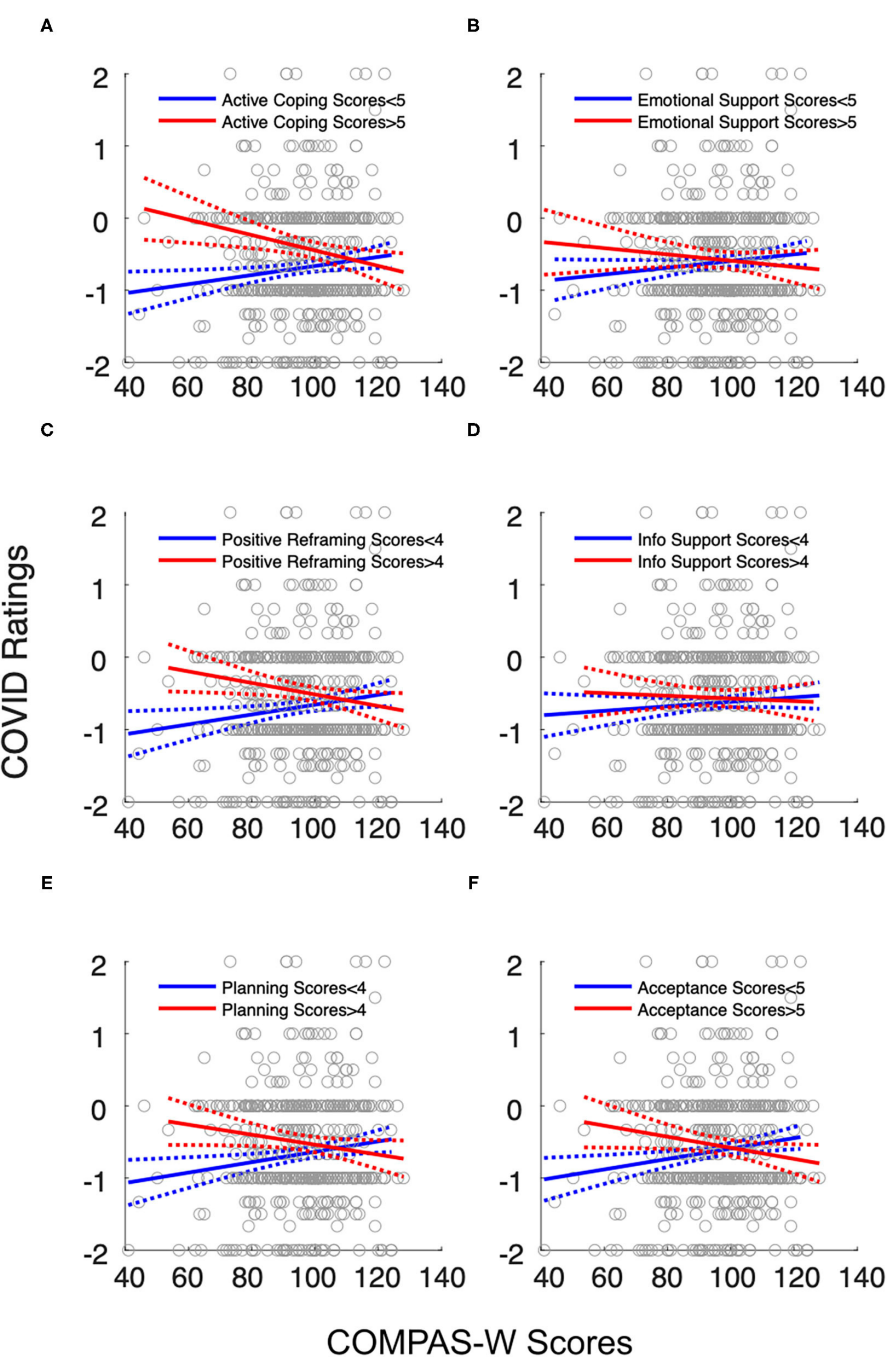
The effect of well-being and specific Approach coping strategies on the impact of COVID isolation on male participants. The blue lines are the least-square regression fits for coping scores below the sample median. The red lines are the least-square regression fits for coping scores above the median. The interactions of COMPAS-W scores and **(A)** active coping, **(C)** positive reframing, and **(E)** planning were significant (*p* < 0.008, Bonferroni-corrected). The interactions of COMPAS-W scores and **(B)** seeking emotional support, **(D)** seeking informational support, and **(F)** acceptance were not significant. Similar to the general Approach coping, specific coping strategies seem to reduce the negative impact of isolation on younger men, but only when their well-being levels are low. Gray dots represent individual scores for COMPAS-W and COVID ratings. Dotted lines represent 95% confidence intervals.

Since Approach coping is a broad strategy containing six specific subscales, we further examined whether specific approach coping strategies were more effective in alleviating the negative impact of isolation on young men than others. Using the Bonferroni-corrected significance level of 0.008 (0.05/6), we found significant interactions of COMPAS-W scores and scores for active coping (β = −0.004, *p* = 0.001; Figure 2A), positive reframing (β = −0.004, *p* < 0.001; Figure 2C), and planning (β = −0.003, *p* = 0.004; Figure 2E), but not for seeking emotional support (β = −0.002, *p* = 0.081; Figure 2B), seeking informational support (β = −0.001, *p* = 0.403; Figure 2D), and acceptance (β = −0.002, *p* = 0.029; Figure 2F). This indicates that certain approach coping strategies are more effective than others in mitigating the negative impact of COVID-19 isolation on young men.

Figure 2 shows that specific Approach coping strategies seem to have a similar pattern with general Approach coping in terms of the effectiveness of reducing negative impacts of COVID-19 isolation on men with low well-being. *Post-hoc* comparisons revealed that isolation ratings were significantly lower in the LCLW group compared to HCLW group for active coping (β = 0.61, *p* < 0.001), positive reframing (β = 0.50, *p* < 0.001), and planning (β = 0.45, *p* < 0.001), but they did not significantly differ between LCHW and HCHW groups (active coping: β = 0.05, *p* = 0.609; positive reframing: β = −0.02, *p* = 0.733; planning: β = −0.10, *p* = 0.283). These results suggest that active coping, positive reframing and planning are effective in reducing the negative impact of COVID-19 separation on men with low levels of mental well-being.

## Discussion

The present study investigated the influence of mental well-being and coping strategies on how COVID-19 isolation affects younger populations. Using a large sample of 1,749 younger Australians and Americans (13–25 years), we found a gender-specific effect on the isolation experience during the pandemic associated with the type of coping strategies and well-being level. Specifically, approach-oriented coping strategies, especially active coping, positive reframing, and planning, appeared to mitigate the negative influence of COVID-19 isolation on young men at low levels of wellbeing. By contrast, there was no evidence of coping strategies having differential effects on the negative experience of COVID-19 isolation for young women. Together, our study suggests that an unexpected stressor such as a pandemic can differentially affect young men and women, especially young men who have low well-being but can actively cope with the stress.

Approach-oriented coping strategies seem to only benefit a specific subset of our sample: young men with lower well-being. By contrast, the effect of approach coping did not manifest in young men who reported having high well-being. This effect is likely the result of the fact that individuals with higher well-being inherently adopt more adaptive coping strategies such as approach coping by default, which does not change regardless of the situation or levels of stress. This is evident by the significant correlations we reported between well-being and different coping strategies. Therefore, individuals who scored high on well-being were likely to use more effective coping strategies, and the benefit may have buffered them from the negative impact of isolation that was reported by others (48–50). On the other hand, this effect was specific to men in the current sample. Therefore, while men and women used similar amounts of approach coping strategies overall, it was only in men with lower wellbeing that the positive benefit of these strategies on COVID-19 isolation were most apparent when these strategies were used more often, as compared to men with lower wellbeing who used the same strategies less often. This effect was specific to COVID-19 related isolation so it would be interesting to replicate the effect in relation to other negative life events. Our next step would be to evaluate whether approach coping strategies promoted increased well-being over time using longitudinal data, and therefore whether this contributes to higher resilience to stressors like the pandemic.

Our results show that male and female participants reported similar negative ratings for COVID-19 isolation, suggesting a similar degree of negative impact of the isolation across men and women. Ostensibly, this contradicts with previous studies reporting that females showed increased distress during the COVID-19 pandemic [(4, 51, 52); but see (53) for evidence of non-specific impacts]. However, a closer look at our data revealed a fine-grained difference in the degree of vulnerability between men and women. This difference stems from the participants who reported lower well-being. Specifically, the impact of COVID-19 isolation on young men with low well-being appeared to be alleviated if they adopted an approach-oriented coping strategy (Figure 1A). By contrast, not using approach coping was associated with more negative feelings about the isolation. This polarized effect is not as extreme for young women, who have a rather homogeneous negative experience regardless of their well-being levels or the coping strategies they rely on (Figure 1B). In other words, young men with low well-being could be more vulnerable to unexpected stressors, particularly if they are less likely to adopt approach coping strategies when dealing with stress.

Deeper analyses revealed that some specific approach coping strategies appeared more effective than others during COVID-19 isolation. Previous studies have shown the effectiveness of actively focusing on a problem in reducing both distress and the likelihood of exhibiting psychiatric symptoms (30, 31, 42, 54, 55). Consistently, our results show that active coping, focusing on the positive side of an otherwise negative experience (i.e., positive reframing), and planning ahead seem effective in reducing the negative impact of COVID-19 isolation. By contrast, we did not find significant effects for other approach-oriented strategies such as seeking informational support. However, actively seeking information has previously been suggested to reduce stress and anxiety during quarantine/isolation (56, 57) and recommended by medical researchers as a preventive measure during lockdown (58). This discrepancy might be attributable to a huge difference in the amount of information easily accessible to the general public. Previous studies were conducted about 15 years ago during the SARS pandemic when information accessibility was far lower than the present time. Actively seeking information was therefore likely to be beneficial in the SARS pandemic but not in the current one. Moreover, the participants in the current study were adolescents and young adults from developed nations, most of whom are savvy at gathering information online, further weakening the effect of effortfully seeking information. Future studies could use populations from underdeveloped regions where the accessibility of information is limited, to examine the role of inadequate information in causing distress during pandemic.

Our results show that adolescents are more negatively influenced by COVID-19 isolation compared to young adults. This aligns with previous findings showing that younger age is a risk factor under stressful circumstances (4, 6, 52, 59), which highlight the challenges faced by adolescents in the COVID-19 pandemic, such as parental stress, risk of abuse, excessive screen time, academic difficulties, and increased tendency for suicide (60–64). Such findings call for extra attention to, and interventions for, this vulnerable group to minimize their risk for mental health problems. Adolescents are more vulnerable to the consequence of isolation presumably because they lack the experience of being independent from parents. Unlike adolescents, young adults in most cases need to attend college and/or work which often entails temporary separations from loved ones such as family and friends. Consequently, they are less likely to be affected by COVID-19 isolation. Living by themselves could also improve their coping skills critical during times of crisis, as shown by the increased use of coping strategies by young adults compared to adolescents we reported here, which aligns with others findings showing age-specific coping styles against the COVID-19 lockdown (65). This might be another reason why young adults appear to fare better than adolescents during the COVID-19 lockdown as they are less reliant on their family for social and financial support.

In addition to gender and age, another demographic factor we considered was country of residence. We did not find evidence of a differential impact of COVID-19 isolation on Australians vs. Americans. However, we note the cross-sectional nature of our study and the timing of it. Our baseline surveys were conducted in May and June when the COVID-19 had been plaguing the two nations for a similar amount of time. In contrast, it was later on during July when the rates of COVID-19 infection reduced significantly in Australia and restrictions in most states were lifted, whereas in the US infection rates have kept skyrocketing beyond the current study's baseline data collection period. Another possible interpretation for the similar ratings is the increased reliance on approach coping strategies by Americans compared to Australians, which is apparent in our results. This might have helped Americans cope with a more severe pandemic. But due to the continuously high infection rates in the US, we speculate that the current results will change to reflect this when we examine longitudinal change over time. Thus, it is important for future studies to examine cross-country differences over time.

A seemingly counterintuitive finding of the current study is that some participants rated the experience of COVID-19 isolation as being positive. The interpretation is likely to be multifaceted. One factor might be a “fear of infection,” which includes both fears about one's own health and the possibility of infecting others (32, 56, 66–69). Previous studies showed that such fear of infection is a major stressor during a pandemic [see (58) for a review]. Separation from loved ones minimizes the chance of infecting them and potentially reduces this fear, which might then account for the positive impact ratings for COVID-19 isolation. Previous studies have also indicated other factors explaining a positive lockdown experience. These include developing new hobbies, increased physical activity, improved sleep quality, greater work flexibility, and calmer life (70, 71). However, we note that the number of participants rating these experiences as being “positive” were significantly less than those rating them as “negative.”

The present study has implications for both clinicians and policy makers. In clinical settings, it is imperative to individualize behavioral therapies and treatments, especially for young men, in accordance with their levels of well-being. This may potentially increase the effectiveness of the treatments as young men seem to respond differentially to active coping mechanisms depending on their ability to maintain a healthy level of well-being under stressful circumstances. This health strategy could be accompanied by policies to emphasize the importance of well-being over and above illness symptoms alone; that is, the importance of living a healthy and balanced life *via* multiple channels such as education and media propaganda, in order to elevate overall well-being in the general population. When future pandemics or adversities arise, policy makers should consider the differential impact of well-being and coping mechanisms on men and women, and prioritize interventions and resources for vulnerable groups, for example, young men, adolescents, and individuals with low levels of well-being.

There are three caveats here. First, the current study focused on adolescents and young adults. Future studies could use older populations and examine whether there is a gender-specific well-being effect on coping effectiveness. Second, the present study is cross-sectional. It would be crucial to systematically examine the longitudinal impact of COVID-19 isolation on mental well-being. A longitudinal design also allows investigating the effectiveness/quality of certain coping strategies over time for promoting well-being. Finally, our study did not include the duration of separation from loved ones when measuring the impact of COVID-19 separation. It would be interesting to examine how the separation duration affects the psychological consequence of COVID-19 lockdown.

In conclusion, using a large sample of 1,749 Australians and Americans, the present study provides cross-sectional evidence that approach-oriented coping strategies can mitigate negative impacts of COVID-19 isolation on young men with low well-being. Our results indicate that approach coping is particularly useful in mitigating isolation distress in young men with lower levels of wellbeing. Our study calls for future research to compare the impact of COVID-19 isolation between men and women over time, and to identity the gender-specific and gender-general predictors for differential outcomes.

## Data Availability Statement

The datasets presented in this article are not readily available due to ethical requirements. Requests to access the datasets should be directed to Justine M. Gatt, j.gatt@neura.edu.au.

## Ethics Statement

This study was reviewed and approved by University of New South Wales HREC (Project number: HC200150). Written informed consent to participate was provided either by the participants if 18 years of age or above, or by the participants' parent/legal guardian if under the age of 18.

## Author Contributions

PC and HP analyzed the data. PC drafted the manuscript. HP and JG assisted with interpretations of the study results and provided revisions to all drafts. JG coordinated the overall project and supervised the research program. All authors contributed to the conceptualization of the current study's aims.

## Conflict of Interest

JG holds stock in Map Biotech Pty Ltd. The remaining authors declare that the research was conducted in the absence of any commercial or financial relationships that could be construed as a potential conflict of interest.

## Footnotes

^1^Humor and Religion are subscales belonging to neither Avoidance nor Approach coping, which are beyond the scope of our study. Therefore, we excluded them from analyses.
